# Optimizing catechin extraction from green tea waste: Comparative analysis of hot water, ultrasound‐assisted, and ethanol methods for enhanced antioxidant recovery

**DOI:** 10.1002/fsn3.4161

**Published:** 2024-04-08

**Authors:** Weerawich Athirojthanakij, Ali Rashidinejad

**Affiliations:** ^1^ School of Food and Advanced Technology Massey University Palmerston North New Zealand; ^2^ Riddet Institute Massey University Palmerston North New Zealand

**Keywords:** antioxidant activity, catechins, extraction yield, green tea waste, hot water extraction, solvent extraction

## Abstract

This study aimed to develop an efficient method for the extraction of bioactive compounds from green tea waste (GTW) toward its potential application in the food industry. GTW, which is generated during the harvesting and processing of green tea products, accounts for a global annual loss of nearly 1 million tonnes. Notably, this waste is rich in polyphenolic compounds, particularly catechins, which are renowned for their significant health benefits. We assessed the optimization of catechin extraction from GTW employing hot water extraction (HWE), ultrasound‐assisted extraction (UAE), and ethanol extraction (EthE) techniques at different sample‐to‐solvent ratios (1:100, 1:50, and 1:20 w/v). The extraction temperature was set at 80°C for both HWE and UAE; however, for EthE, the temperature was slightly lower at 70°C, adhering to the boiling point of ethanol. High‐performance liquid chromatography was used to determine the extraction efficiency by quantifying various catechins (i.e., catechin, epicatechin [EC], epicatechin gallate [ECG], epigallocatechin [EGC], and epigallocatechin gallate [EGCG]). In terms of the concentration for individual catechins, EC was found to be the highest concentration detected, ranging from 30.58 ± 1.17 to 37.95 ± 0.84 mg/L in all extraction techniques and ratios of solvents, followed by EGCG (9.71 ± 1.40–20.99 ± 1.11 mg/L), EGC + C (7.95 ± 0.66–12.58 ± 0.56 mg/L), and ECG (1.85 ± 0.71–6.05 ± 0.06 mg/L). The findings of DPPH (2,2‐diphenyl‐1‐picryl‐hydrazyl) free radical assay illustrated that HWE demonstrated the highest extraction efficiency at all ratios, ranging from 61.41 ± 1.00 to 70.36 ± 1.47 mg/L. The 1:50 ratio exhibited the highest extraction yield (25.98% ± 0.75%) compared to UAE (24.16% ± 0.95%) and EthE (22.59% ± 0.26%). Moreover, this method of extraction (i.e., HWE) produced the highest total catechins and %DPPH reduction. Consequently, HWE was the most efficient method for extracting catechins from GTW, underscoring its potential for valorizing waste within the food manufacturing industry.

## Graphical abstract

This study explored the optimization of catechin extraction from green tea waste (GTW) using hot water extraction (HWE), ultrasound‐assisted extraction (UAE), and ethanol extraction (EthE) techniques at different sample‐to‐solvent ratios. HWE demonstrated the highest extraction efficiency across all ratios, with the 1:50 ratio exhibiting the highest extraction yield compared to UAE and EthE. The findings highlight HWE as the most efficient method for extracting catechins from GTW, highlighting its potential for waste valorization in the food manufacturing industry.

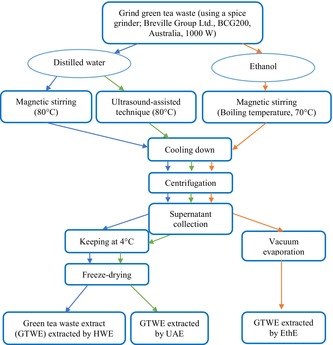

**Practical application**

This research highlights the potential for utilizing green tea waste, a by‐product of tea processing, as a valuable source of health‐promoting bioactive compounds, specifically catechins. The study demonstrates that employing hot water extraction (HWE) at specific conditions yields the highest efficiency in extracting catechins, presenting an environmentally friendly and economically viable approach for the food manufacturing industry to recover antioxidants from abundant green tea waste. The application of this optimized extraction technique could aid in fostering sustainable practices, such as reducing water and energy consumption, minimizing waste, and utilizing health‐enhancing compounds for potential industrial applications.

## 1 INTRODUCTION

Green tea (*Camellia sinensis*), which is a source of bioactive compounds such as catechins, flavonoids, fluoride, and flavonol glycosides (Graham, 1992; Yuwono, 2005), is produced from green tea leaves without fermentation (Ho et al., 2009; Rashidinejad et al., 2014). Normal and high qualities of green tea leaves are transferred to the manufacturing processes. The first process is steaming the green tea leaves for 45–60 s, in order to inactivate endogenous enzymes. If the enzymes are not deactivated, they can lead to the fermentation process (The oxidation of catechins) (Ho et al., 2009; Willson & Clifford, 1992). To extend the shelf‐life of green tea leaves, the drying process, considered as the process generating the highest green tea waste, is indispensable to decrease the moisture content. To achieve the desired moisture content (about 6%) for the desirable storage of tea leaves, there are three drying stages (depending on the temperature and time). First, tea leaves are curled and dried in hot air at 90–110°C for 40–50 min, to decrease the moisture content from 76% to 50%. Second, green tea leaves are pressed and dried at 50–60°C for 30–40 min to reduce the moisture to 30%. Last, the goal of 6% moisture content is accomplished by drying the tea leaves at 80–90°C for 40 min (Willson & Clifford, 1992). The final stage in green tea manufacturing is the refining process and quality screening. It is during this final process that the leaves with poor appearance and disunity (known as crude tea leaves) are separated to remove dust and sold as a low‐quality tea product or tea waste; whereas, the purified leaves are packed as a high‐quality tea product (Ho et al., 2009; Willson & Clifford, 1992).

Several green tea products have been developed for human consumption. However, the production of green tea still generates a substantial amount of waste during both the harvest and processing stages (Wang et al., 2012, 2016). Currently, this waste is amounted to approximately 5.8 million tons, and there are indications that this quantity is on an upward trajectory (Ma et al., 2022; Wang et al., 2012, 2016). Huge amounts of tea waste are converted into activated carbon, fertilizer, or feed additives. To maximize the benefits of such a waste, one of the most effective ways is to extract its bioactive compounds, which have the potential to provide health‐promoting properties in food and nutraceutical products (Sui et al., 2019).

Fresh green tea leaves retain 36% polyphenols, dominated by catechins. Green tea catechins can be divided into (−)‐epigallocatechin‐3‐gallate (EGCG) (which provides the highest potential of antioxidant activity, when compared to other catechin derivatives), (+)‐catechin (C), (−)‐epicatechin‐3‐gallate (ECG), and (−)‐epigallocatechin (EGC) (Choung et al., 2014; Du et al., 2012; Kim et al., 1994; Koch et al., 2020; Rice‐Evans, 1999; Roccaro et al., 2004; Song et al., 2005). EGCG is predominantly found in fresh green tea leaves at the percentages of 48%–55% of total polyphenols, possessing five times greater antioxidant capacity than vitamins C and E (Cabrera et al., 2003; Rice‐Evans, 1999; Saffari & Sadrzadeh, 2004). In the aspect of health‐promoting properties, green tea catechins play a crucial role in the increment of an antioxidant level and the reduction in oxidative stress, and accordingly, the reduction in the risk of chronic diseases such as cardiovascular, cancer, neurodegenerative, and Alzheimer's diseases (Lorenzo & Munekata, 2016; Xing et al., 2019).

Green tea bioactives are generally obtained by different extraction methods including hot water extraction (HWE), ethanol extraction (EthE), maceration, infusion, organic solvent extraction, supercritical carbon dioxide extraction, microwave‐enhanced vacuum extraction, and ultrasound‐assisted extraction (UAE) (Chang et al., 2000), soxhlet extraction, and ultra‐high‐pressure extraction (Jun et al., 2011). Extraction parameters such as the variation of solvent type, pH, temperature, time, and the ratio of green tea to solvents have a direct effect on the extraction efficiency and yield of green tea extract (Kim et al., 2008; Lou et al., 2012; Perva‐Uzunalić et al., 2006; Tang et al., 2010; Wang & Helliwell, 2000; Yoshida et al., 1999). The HWE is the easiest method to isolate catechins from green tea; however, the proper extraction condition needs to be controlled. Perva‐Uzunalić et al. (2006) reported that the best temperature (and time) that could provide the highest catechin content was 80°C for 20 min. Vuong et al. (2010) also reported that the most efficient condition for catechin extraction via hot water was 80°C for 30 min. Liang et al. (2007) found that the EGCG concentration extracted from 50% ethanol was 26.3% higher than hot water extraction at 80°C. There are several studies demonstrating that ultrasound‐assisted extraction is an efficient method and is considered a green extraction technology for the extraction of green tea catechins. Koch et al. (2020) reported that a higher catechin concentration was found in UAE (340.53 mg/200 mL) than in HWE (326.94 mg/200 mL).

Concerning scalability and application in the food industry, HWE is a scalable method that can be easily implemented on an industrial scale. Thus, its potential for scaling up feasibility aligns well with exploring practical and efficient approaches in food processing. It offers several advantages, including cost efficiency, safety, environmental friendliness, and high extraction efficiency. The UAE is also a viable method for industrial production. Its suitability stems from several factors. Firstly, it enables the extraction of heat‐sensitive compounds without compromising their availability (Patist & Bates, 2008). Secondly, it enhances extraction yield, resulting in improved efficiency (Wen et al., 2018). Lastly, it reduces the extraction time (Patist & Bates, 2008; Wen et al., 2018). In a study conducted by Tamminen et al. (2022), the scaling up of UAE in the extraction of spinach leaves was examined, specifically focusing on the extraction yield of chlorophylls and carotenoids. The findings revealed that pilot‐scale UAE (using a frequency of 25 kHz and power density of 1500 W/L) achieved a 1.9‐fold increase in extraction yield compared to conventional solvent extraction methods and yielded nearly identical results to laboratory‐scale UAE. However, the successful scaling up of UAE necessitates the expert design of the extraction system, encompassing ultrasound probes, extraction tanks, and agitation systems, to ensure consistent extraction efficiency and yield.

Accordingly, this study was designed with the objective of devising an efficient method for extracting bioactive compounds from GTW, with a view toward its potential utilization in the food industry. We sought to evaluate different extraction methods, including HWE, UAE, and EthE, to identify the method that delivers the highest efficiency and yield. We hypothesized that by comparing these three methods, the study would highlight the most effective and efficient extraction technique. This information would be of value to researchers and industries interested in harnessing GTW as a source of bioactive compounds. Furthermore, the findings of this study may pave the way for the development of novel techniques for extracting bioactive compounds from other natural sources.

## 2 MATERIALS AND METHODS

### 2.1 Green tea waste, reagents, and chemicals

Dried green tea waste (Guiding Niaowang) was obtained from Guizhou Eight Grams Tea and Agricultural Development Ltd. (Qiannan, China). The green tea waste sample used in the study contained fine particles in granular shape with vibrant green color, as well as some finer particles with a fluffy texture. The catechin standards of high purity (≥97.0% purity, HPLC grade) including catechin (C), epicatechin (EC), epicatechin gallate (ECG), epigallocatechin (EGC), and epigallocatechin gallate (EGCG) were purchased from Sigma‐Aldrich, Inc. (Darmstadt, Germany). 2,2‐Diphenyl‐1‐picryl‐hydrazyl (DPPH) reagent was procured from Sigma‐Aldrich Co., Inc. (Darmstadt, Germany). All other chemicals and reagents used in this study were of analytical grade.

### 2.2 Extraction of green tea waste catechins

**FIGURE 1:**
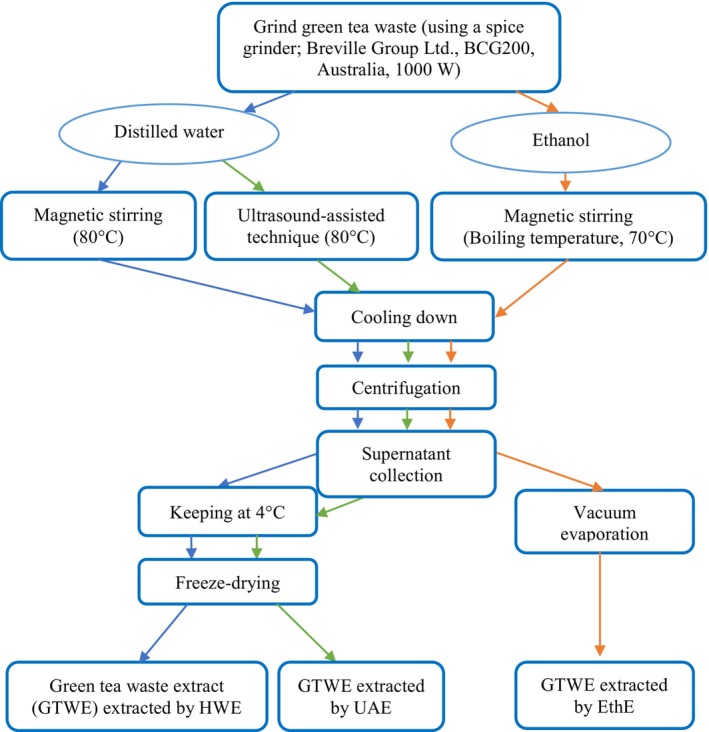
Extraction processes applied in the current study. (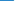) Hot water extraction (HWE), (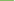) ultrasound‐assisted extraction (UAE), and (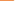) ethanol extraction (EthE).

The extraction method of GTW was based on the methods developed by Choung et al. (2014) and Perva‐Uzunalić et al. (2006), with specific modifications for this study. A schematic representation of the laboratory‐scale extraction process is illustrated in Figure 1. Initially, the dried green tea waste leaves were finely ground using a Breville grinder (Breville Group Ltd., Auckland, New Zealand) and sieved through a 0.425 mm screen to achieve uniform particle size, facilitating efficient extraction. The resulting particles were then carefully stored in airtight plastic bags at 4°C until further use to preserve the integrity of the polyphenolic compounds.

For the hot water extraction (HWE) and ultrasound‐assisted extraction (UAE) methods, distilled water was used as the solvent. The HWE method involved subjecting the ground green tea waste to an extraction temperature of 80°C for 20 min. In the case of UAE, the sample was exposed to ultrasonic waves during the extraction process at the same temperature (80°C), frequency (20 kHz), power (120 W), and time (20 min). These conditions were chosen to ensure optimal extraction of the target polyphenolic compounds, particularly catechins, while minimizing the degradation of heat‐sensitive components. On the other hand, ethanol extraction (EthE) was carried out using 80% ethanol as the extracting solvent, with the extraction temperature set at 70°C, corresponding to the boiling temperature of ethanol, for the same duration of 20 min. The lower temperature was selected to prevent excessive degradation of the sensitive compounds during the extraction process, while still promoting efficient extraction.

Post‐extraction, to preserve the integrity of the extracted compounds, the samples were promptly stored at −20°C overnight to inhibit enzymatic reactions and oxidation. Subsequently, the samples underwent freeze‐drying in a Cuddon FD18 Freezer Drier (manufactured by Cuddon Freeze Dry, Blenheim, New Zealand) under controlled conditions of 1 mbar pressure and a duration of 72 h. This freeze‐drying process was crucial for removing residual moisture from the samples without causing degradation, thereby ensuring the stability and preservation of the catechins for further analysis and evaluation.

### 2.3 Extraction efficiency

#### 2.3.1 High‐performance liquid chromatography (HPLC) analysis

Catechins were analyzed based on the method reported by Wang et al. (2003) with slight modifications. An Agilent 1200 series HPLC machine equipped with a C18 reversed phase Synergi 4 μm (150 × 4.6 mm) was used. The mobile phases were composed of eluent A (containing 0.1% orthophosphoric acid in the water, v/v) and eluent B (containing 0.1% orthophosphoric acid in methanol, v/v). The gradient was set as 0–5 min, 20% B; 5–7 min, linear gradient from 20 to 24% B; 7–10 min, 24% B; 10–20 min, linear gradient from 24% to 40% B; 20–25 min, linear gradient from 40% to 50% B. Post‐run time was 1 min, the flow rate of the eluents was controlled at 0.8 mL/min, and the injection volume was 5 μL. The column temperature was kept at 30°C. In terms of detection, a diode array detector was recorded at 210 and 280 nm. The identification of catechins was based on the retention times of their peaks and UV–vis spectra compared with the calibration curve made from standards (2–50 mg/L) of each catechin, including catechin, epicatechin, epicatechin gallate, epigallocatechin, and epigallocatechin gallate.

#### 2.3.2 Total antioxidant activity

DPPH (2,2‐diphenyl‐1‐picryl‐hydrazyl) free radical method was applied following the method of Kara and Erçelebi (2013) with some modifications. The DPPH solution was prepared fresh daily by dissolving DPPH 1.2 mg with methanol 50 mL. The prepared liposome samples were diluted at the ratio of 1:10 (v/v) with methanol, and then 0.1 mL of the prepared samples were mixed with 3.9 mL of DPPH solution. The mixtures were kept in the dark at room temperature for 30 min. After that, the absorbance was determined by spectrophotometer (GENESYS 10 Series, UV‐vis, USA) at 515 nm.

The %DPPH reduction was calculated using the formula below (Equation 1):

(1) $$\%\text{DPPH reduction}=\left(\;\frac{A_{c}-A_{s}}{A_{c}}\;\right)\times 100$$

where, AC = absorbance of the control (*t* = 0 min), AS = the absorbance of a tested sample at the end of the reaction (*t* = 30 min).

All measurements were carried out at room temperature (25°C). The %DPPH of the samples was compared with the calibration curve made using (+)‐catechin as the standard (0.20–25 mg/L) (Chen et al., 2013).

### 2.4 Statistical analysis

The reported data are means of at least three measurements. All measurements were performed in triplicates. Minitab (Version 17.3.1) statistical software (Minitab Inc., State College, PA) was used for carrying out the corresponding statistical analysis. The data were subjected to the analysis of variance (ANOVA) for the mean comparison for any significant differences (*p* < .05). All graphical presentations were generated by Microsoft Excel 2016 (Microsoft Corporation, Redmond, WA).

## 3 RESULTS AND DISCUSSION

### 3.1 The effect of extraction techniques on the extraction yield

The extraction yield (EY) values reflect the catechin extraction efficiency because a higher EY leads to a lower production cost (Pasrija & Anandharamakrishnan, 2015). The application of extraction methods has a direct consequence on EY; thus, three different techniques, consisting of HWE, UAE, and EthE, were implemented in the case of this study to demonstrate the most efficient extraction technique for the extraction of green tea catechins from GTW.

As seen in Table 1, the extraction techniques and GTW:solvent ratio (w/v) affected the EY of the individual samples differently. Among the three extraction techniques, UAE showed the highest yield at the ratio of 1:100 (29.93% ± 1.21%) and 1:20 (20.64% ± 1.45%), while HWE provided the highest results at the ratio of 1:50 (25.98% ± 0.75%). The lowest EY belonged to EthE in any applied ratio. The differences among these obtained values can be attributed to the difference in the extraction techniques and the frequency of an ultrasonic wave. Perva‐Uzunalić et al. (2006), who studied the influence of different extraction techniques (HWE and EthE) on the extraction efficiency, also reported that HWE (80°C, 20 min) gained slightly higher EY than EthE (boiling temperature, 20 min) at about 36.5% and 34.5%, respectively. Furthermore, Albuquerque et al. (2017), who conducted a study to optimize the extraction methods for catechins from *Arbutus unedo L*. fruits, found that microwave extraction yielded the highest amount of catechin (1.70 ± 0.3 mg/g of dry weight [dw]) under conditions of 42.2 ± 4.1 min, 137.1 ± 8.1°C, and a solvent mixture of 12% ethanol and 88% water. Maceration extraction followed, yielding 1.38 ± 0.1 mg/g dw of catechin at 93.2 ± 3.7 min and 79.6 ± 5.2°C, and a solvent mixture of 24% ethanol and 76% water. UAE exhibited the lowest yield, 0.71 ± 0.1 mg/g dw of catechin, under conditions of 42.4 ± 3.6 min and 314.9 ± 21.2°C, and a solvent mixture of 40% ethanol and 60% water.

**TABLE 1:** Effect of various extraction techniques and the ratio of green tea waste (GTW) to solvents (w/v) on the extraction yield (%) of catechins in GTW.

Extraction technique	Extraction yield (%)^†^		
	1:100^‡^	1:50^‡^	1:20^‡^
Hot water (HWE)	29.30 ± 1.21^ab^	25.98 ± 0.75^a^	18.92 ± 1.45^a^
Ultrasound‐assisted (UAE)	29.93 ± 0.59^a^	24.16 ± 0.95^b^	20.64 ± 0.51^a^
Ethanol (EthE)	27.32 ± 0.34^b^	22.59 ± 0.26^b^	18.46 ± 0.27^a^

*Note*: Values with different superscripted letters within the same column are significantly different (*p <* .05).

^†^ Values are the mean of three replications (*n* = 3 ± SD).

^‡^ The ground green tea waste to solvents ratio (w/v).

Regarding the effect of ultrasound frequency, Afroz Bakht et al. (2019) reported similar results for the difference in extraction techniques, consisting of EthE and UAE, and ultrasonic frequency of branded tea samples (e.g., Lipton, Rabea, Alkbous, Green Gold, and Haritham) that affected the EY. In the current experiment, the EY of EthE was about 12.35%, which was significantly lower than UAE with a frequency of 26 kHz (about 18.61%). The variation in the ultrasonic frequency also played an essential role in the EY of the samples in this study. The highest EY belonged to 40 kHz (18.61%), followed by 50 kHz (14.89%) and 30 kHz (10.78%). Due to the frequency limitation of the sonicator probe, the highest frequency of UAE that was implemented in this experiment was 20 kHz, which is lower than the minimum frequency (30 kHz) applied in the study of Afroz Bakht et al. (2019). This could have negatively affected the EY of UAE samples because the yield of UAE was not significantly different (*p* < .05) from HWE at the ratio of either 1:100 or 1:20. However, HWE gained significantly (*p* < .05) higher EY (25.98% ± 0.75%) at the ratio of 1:50. In summary, HWE provided the highest EY over both UAE and EthE at the ratio of 1:50, but there was no significant difference (*p* < .05) in the case of 1:100 and 1:20 ratios. Therefore, it can be said that the UAE method with low frequency (20 kHz) could not provide a higher EY than other extraction techniques. Lastly, although both HWE and EthE are similar in principle, they are different in terms of the solvents used and the compounds they can extract. HWE uses hot water to dissolve and extract water soluble compounds, while EthE, on the other hand, uses ethanol, a polar solvent, which can dissolve a wider range of compounds, including those not soluble in water. Thus, the choice of solvent determines the types of compounds that are extracted and the potential uses of the extracted material.

### 3.2 The effect of extraction techniques on catechin extraction efficiency

The concentration of the extracted green tea catechins relates to the extraction methods, as the first step for the separation of these bioactive compounds from GTW (Choung et al., 2014; Perva‐Uzunalić et al., 2006; Tiwari, 2015). In this study, five types of catechins, consisting of C, EGC, EGCG, EC, and ECG, were analyzed via HPLC and DPPH methods, to find the most efficient extraction technique and extraction condition for the separation of catechins from GTW.

**FIGURE 2:**
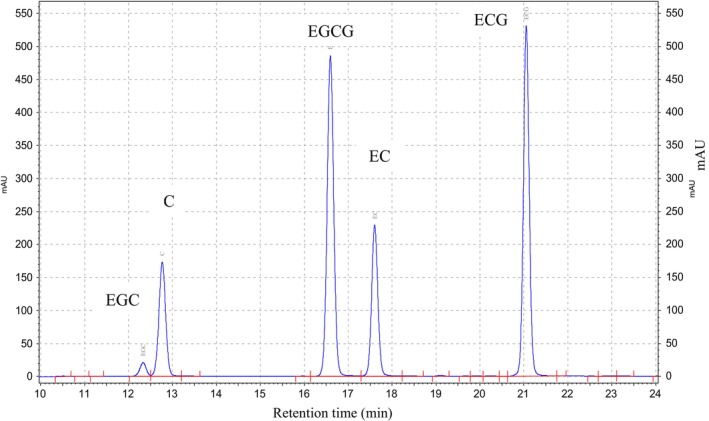
Chromatogram of the standard catechin compounds at the concentration of 500 ppm, analyzed by high‐performance liquid chromatography (HPLC). C, (+)‐catechin; EC, (−)‐epicatechin; ECG, (−)‐epicatechin gallate; EGC, (−)‐epigallocatechin; EGCG, (−)‐epigallocatechin gallate.

**FIGURE 3:**
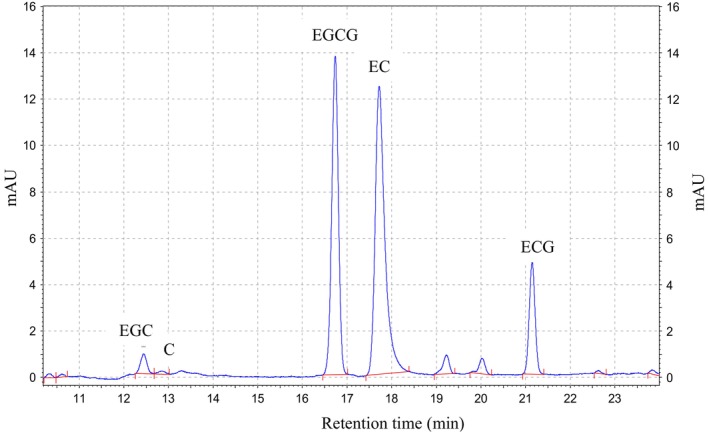
Chromatogram of the green tea waste extract analyzed by HPLC. C, (+)‐catechin; EC, (−)‐epicatechin; ECG, (−)‐epicatechin gallate; EGC, (−)‐epigallocatechin; EGCG, (−)‐epigallocatechin gallate. *Note*: two of the peaks (unmarked) are not known.

The HPLC chromatograms of the standard compounds and the green tea waste extract samples are provided in Figures 2 and 3, respectively. Peak separation and integrations were implemented based on the guidelines provided by Lee and Ong (2000). The concentration results of five catechins are also presented in Table 2. Based on these findings, the ratio of 1:100 demonstrated the highest total catechins at the range of 66.37 ± 1.27–70.36 ± 1.47 mg/L, followed by 1:50 and 1:20 at 62.57 ± 0.65–67.83 ± 0.83 mg/L and 56.57 ± 1.90–61.41 ± 1.00 mg/L, respectively. Total catechins from HWE possessed the highest catechin concentration with statistical significance (*p* < .05) in the case of every ratio (i.e., 1:100, 1:50, and 1:20), followed by UAE and EthE. In terms of the values for individual catechins, EC was found to be the highest concentration detected, ranging from 30.58 ± 1.17 to 37.95 ± 0.84 mg/L in all extraction techniques and ratios of solvents, followed by EGCG (9.71 ± 1.40–20.99 ± 1.11 mg/L), EGC + C (7.95 ± 0.66–12.58 ± 0.56 mg/L), and ECG (1.85 ± 0.71–6.05 ± 0.06 mg/L). In summary, the arranged order of the individual catechins in GTW was EC > EGCG > EGC + C > ECG. It is notable that the reason for reporting EGC together with C (i.e., EGC + C) in this study is that the concentration of C was considered low, and the peak of C was not completely separated from EGC (Figure 3); nevertheless, such a combination of peaks/data did not affect the data evaluation.

**TABLE 2:** Effect of various extraction techniques and the ratio of green tea waste to solvents (w/v) on the concentration (mg/L) of catechins in this by‐product, analyzed by HPLC.

Extraction technique	Concentration (mg/L)^†^				
	EGC + C	EGCG	EC	ECG	Total catechins
1:100^‡^					
Hot water (HWE)	12.58 ± 0.56^a^	20.99 ± 1.11^a^	30.74 ± 0.60^b^	6.05 ± 0.06^a^	70.36 ± 1.47^a^
Ultrasound‐assisted (UAE)	10.93 ± 1.19^ab^	16.75 ± 1.41^b^	35.38 ± 2.39^a^	5.36 ± 0.42^ab^	68.42 ± 0.55^ab^
Ethanol (EthE)	10.00 ± 0.38^b^	13.19 ± 0.48^c^	37.95 ± 0.84^a^	5.23 ± 0.29^b^	66.37 ± 1.27^b^
1:50^‡^					
Hot water (HWE)	12.01 ± 0.93^a^	19.73 ± 0.50^a^	31.49 ± 1.09^b^	4.62 ± 0.49^ab^	67.83 ± 0.83^a^
Ultrasound‐assisted (UAE)	11.73 ± 1.23^a^	17.55 ± 0.90^b^	30.58 ± 1.17^b^	3.70 ± 0.43^b^	63.56 ± 1.08^b^
Ethanol (EthE)	8.65 ± 0.47^b^	13.11 ± 0.55^c^	35.81 ± 0.44^a^	5.00 ± 0.18^a^	62.57 ± 0.65^b^
1:20^‡^					
Hot water (HWE)	10.34 ± 0.51^a^	14.60 ± 0.88^a^	32.65 ± 0.76^b^	3.83 ± 0.54^a^	61.41 ± 1.00^a^
Ultrasound‐assisted (UAE)	9.31 ± 0.69^ab^	9.71 ± 1.40^b^	35.71 ± 0.37^a^	1.85 ± 0.71^b^	56.57 ± 1.90^b^
Ethanol (EthE)	7.95 ± 0.66^b^	10.78 ± 0.66^b^	34.14 ± 1.07^ab^	4.11 ± 0.55^a^	56.97 ± 0.49^b^

*Note*: Values with different superscripted letters within the same column are significantly different (*p <* .05).

Abbreviations: C, (+)‐catechin; EC, (−)‐epicatechin; ECG, (−)‐epicatechin gallate; EGC, (−)‐epigallocatechin; EGCG, (−)‐epigallocatechin gallate.

^†^ Values are the mean of three replications (*n* = 3 ± SD).

^‡^ The ground green tea waste to solvents ratio (w/v).

Koch et al. (2018) studied the effect of green tea origin on the quantity of individual catechins and suggested that the catechin concentrations of green tea originating from Sri Lanka, Japan, and South Korea could be placed in the following order: EGC > EGCG > ECG > EC > C, while green tea coming from China, India, and Nepal presented a different order; that is, EGCG > EGC > ECG > EC > C. Moreover, EGC and EGCG were the most dominant catechins in all sources of green tea, accounting for 65%–80%. In another study, Henning et al. (2003) reported the content of catechins in 18 commercial tea products. Five catechins from these commercial products were presented from the highest to the lowest quantity. For instance, “Lipton” green tea contained catechins in the order of EGCG > EGC > ECG > EC > C at concentrations of 83.9, 76.4, 13.7, 11.9, and 5.8 mg/100 mL, respectively. “Bigelow” green tea consisted of EGCG > EGC > EC > ECG > C at the concentrations of 42.5, 30.9, 6.5, 3.6, and 0 mg/100 mL, respectively. For these reasons, it could be concluded that the different sources and brands of green tea (reflecting different processing methods) play a crucial role in the quantity and proportion of catechins. In the case of the current experiment, the results of HPLC analysis corresponded well with the findings of Koch et al. (2018) and Henning et al. (2003), where HWE was considered the most efficient extraction technique compared with UAE and EthE, because HWE samples contained the highest amount of catechins in every ratio of the solvents. A study conducted by Ayyildiz et al. (2018) compared the extraction efficiency of green tea catechins using HWE between a small laboratory scale and an industrial pilot scale. The extraction process involved applying a temperature of 85°C for 30 min with a tea: water ratio of 1:25. The results revealed that the concentration of catechins, namely EGCG, EGC, ECG, and EC, in both scales of extractions were similar (EGCG: 3.810 ± 0.26 g/100 g, EGC: 2.290 ± 0.11 g/100 g, ECG: 0.649 ± 0.03 g/100 g, and EC: 0.412 ± 0.02 g/100 g).

DPPH free radical scavenging method was applied to determine the antioxidant capacity of GTW samples and to confirm the efficacy of various extraction methods. Table 3 shows the antioxidant capacities of GTW treated with different extraction techniques and the various ratios of solvents. The highest total catechins and %DPPH reduction were found in the case of HWE, while the antioxidant performance of UAE was not significantly different than that of EthE. However, at the ratio of 1:100, there was no significant difference (*p* > .05) among all methods in terms of total catechins and %DPPH reduction. This might be attributed to the chemical stability of catechins in GTWE under different conditions. Recently, Koch et al. (2020) studied the effect of extraction solvents (ethanol and water) and extraction techniques (HWE, UAE, and EthE) on the antioxidant capacity of green tea samples (from Sri Lanka) using the DPPH method. All extraction methods were kept constant in terms of temperature and time (80°C and 10 min) and the frequency of UAE was set at 45 kHz. The results demonstrated that UAE gained the lowest antioxidant activity (59.89%) compared with both HWE and EthE (66.64% and 75.08%, respectively). The reason for the lowest antioxidant activity of UAE was speculated to be associated with the applied ultrasonic wave that could have negatively affected the chemical stability of catechins (via degradation and/or isomeration), leading to lower values for DPPH antioxidant activity. Additionally, the ultrasonic wave may have a negative effect on the bioactivity of green tea catechins in specific frequencies; for example, in the case of 45 kHz that was implemented by Koch et al. (2020). These effects could be due to the degradation of catechins under the influence of ultrasonic waves at this specific frequency, which could potentially lead to a decrease in the antioxidant activity of the green tea extract. It is important to note that the effects of ultrasonic waves on the bioactivity of green tea catechins are complex and can be influenced by various factors, including the specific conditions of the extraction process (such as temperature and duration), the concentration of the solvent used, and the specific type of green tea used. Therefore, further research is needed to fully understand these effects and to optimize the use of ultrasonic waves in the extraction of bioactive compounds from GTW.

**TABLE 3:** Effect of various extraction techniques and the ratio of the ground green tea waste (GTW) to solvents (w/v) on total catechin concentration (catechin equivalent) and %DPPH reduction in GTW catechins analyzed by DPPH free radical scavenging method.

Extraction technique	%DPPH reduction^†^		
	1:100^‡^	1:50^‡^	1:20^‡^
Hot water (HWE)	35.88 ± 0.66^a^	35.36 ± 1.44^a^	33.26 ± 1.38^a^
Ultrasound‐assisted (UAE)	34.01 ± 1.61^a^	31.94 ± 1.44^b^	30.12 ± 0.52^b^
Ethanol (EthE)	33.37 ± 1.50^a^	32.18 ± 0.70^b^	30.11 ± 0.72^b^

*Note*: Values with different superscripted letters within the same column are significantly different (*p <* .05).

^†^ Values are the mean of three replications (*n* = 3 ± SD).

^‡^ The ground green tea waste to solvents ratio (w/v).

Using another antioxidant activity assay (oxygen radical absorption capacity assay [ORAC]), Sun et al. (2015) reported that ultrasound waves improved the extraction of polyphenols, but later free radicals that were produced by sonification increased the degradation of these bioactive compounds. In contrast, several other studies presented that the ultrasonic wave would be beneficial for the extraction of polyphenols because of the endorsement of the heat‐sensitive compound extraction and other advantages such as clean energy, green energy, organic solvent rejection, enhancement of extraction yield (EY), and the development of extraction performance (Choung et al., 2014; Tiwari, 2015). For example, according to Muzaffar et al. (2016), when an ultrasonic wave of 33 kHz with different treatment times (0, 10, 20, 30, 40, and 60 min) was applied to extract the polyphenols from cherries, %DPPH reduction analysis showed that the longer extraction time resulted in higher antioxidant activity. However, several various factors could negatively affect the extraction efficiency of polyphenols; pH, temperature, time, and characteristics of the food (Koch et al., 2020).

Regarding the implementation of UAE in the pilot plant scale, Saklar et al. (2018) reported that the UAE had a lower potential to extract all kinds of catechins from green tea leaves than HWE. The extraction condition of HWE was fixed at 85°C for 30 min while the extraction time and temperature of UAE were performed at 70 and 80°C for 30 and 60 min. HPLC results of all catechins indicated that HWE was better than UAE. For example, the concentration of EGCG extracted from HWE was 3.81 g/100 g dw, which was significantly higher than every extraction condition of UAE (1.74–2.46 g/100 g dw). Therefore, HWE gains a higher possibility to scale up at the pilot plant scale, but more investigation is to be carried out in the case of UAE, in terms of ultrasonic probes, extraction tank, and agitation system.

Generally speaking, solvent extraction is widely recognized for its ability to extract phytochemical substances on a large scale, as it requires fewer solvents and consumes less energy while still yielding high amounts of desired compounds (Zia et al., 2022). Additionally, solvent extraction has undergone advancements through the utilization of novel techniques such as microwave‐assisted extraction, supercritical fluid extraction, pressurized liquid extraction, and UAE (Chuo et al., 2022; Lefebvre et al., 2021). These techniques aim to optimize extraction efficiency and yield while also minimizing extraction costs. However, it is important to note that solvent extraction generates chemical waste, which will require thorough treatment processes to ensure environmental sustainability and prevent any adverse impacts on the ecosystem (Kirtane et al., 2022). Therefore, by addressing the scalability and feasibility aspects of water extraction and its comparison with other methods of extraction, this research contributes to the broader discourse on optimizing food processing techniques for industrial applications. Nonetheless, when it comes to the scale‐up of any process in the food industry, the importance of expert design and optimization for achieving consistent extraction efficiency and yield cannot be undervalued.

## 4 CONCLUSIONS

Taken together, this study highlights the importance of GTW as a substantial source of catechins. The data demonstrated HWE's superiority in terms of extraction yield and efficiency compared to UAE and EthE across most of the tested ratios. For example, the concentration of EGCG obtained through HWE was much higher than the concentrations achieved through all UAE conditions (3.81 vs. 1.74–2.46 g/100 g of dry weight). Hence, HWE emerges as the most effective method for extracting potent antioxidant compounds of GTW. It is a straightforward and economical technique, requiring fewer steps and equipment than alternative methods, making it more accessible for food manufacturers. Furthermore, HWE's gentle extraction process minimizes the risk of contamination with undesired residues or impurities, and catechin degradation. Notably, HWE exhibited the highest total catechins and %DPPH reduction. In addition to its simplicity and cost‐effectiveness, HWE is an environmentally friendly approach, avoiding the production of hazardous waste and excessive energy consumption. However, the increased use of UAE compared with other innovative extraction techniques necessitates further investigation, particularly for extracting bioactives from agricultural waste materials. These findings offer crucial insights into the most efficient pathways for extracting catechins from GTW, which could significantly impact the food and nutraceutical sectors. Therefore, green tea producers should consider the valorization of GTW to promote its sustainable and effective utilization. This could lead to significant advancements in the industry and contribute to a more sustainable future.

## AUTHOR CONTRIBUTIONS

**Weerawich Athirojthanakij:** Conceptualization (equal); data curation (lead); formal analysis (equal); investigation (lead); methodology (lead); project administration (equal); software (lead); visualization (lead); writing – original draft (lead). **Ali Rashidinejad:** Conceptualization (equal); funding acquisition (lead); investigation (equal); methodology (equal); project administration (equal); resources (lead); supervision (lead); validation (lead); writing – review and editing (lead).

## CONFLICT OF INTEREST STATEMENT

The authors declare that they have no conflict of interest. This research did not receive any specific grant from funding agencies in the public, commercial, or not‐for‐profit sectors.

## Data Availability

The data that support the findings of this study are available on request from the corresponding author.
